# Trends and Challenges of SPR Aptasensors in Viral Diagnostics: A Systematic Review and Meta-Analysis

**DOI:** 10.3390/bios15040245

**Published:** 2025-04-12

**Authors:** Elba Mauriz

**Affiliations:** 1 Department of Nursing and Physiotherapy, Universidad de León, Campus de Vegazana, s/n, 24071 León, Spain; elba.mauriz@unileon.es; Tel.: +34-987-293617; 2 Institute of Food Science and Technology (ICTAL), La Serna 58, 24007 León, Spain

**Keywords:** surface plasmon resonance, SPR, biosensor, aptamer, virus, diagnostics, aptasensor

## Abstract

Surface plasmon resonance (SPR) aptasensors benefit from the SPR phenomenon in measuring aptamer interactions with specific targets. Integrating aptamers into SPR detection enables extensive applications in clinical analysis. Specifically, virus aptasensing platforms are highly desirable to face the ongoing challenges of virus outbreaks. This study systematically reviews the latest advances in SPR aptasensors for virus detection according to PRISMA guidelines. The literature search recovered 322 original articles from the Scopus (n = 152), Web of Science (n = 83), and PubMed (n = 87) databases. The selected articles (29) deal with the binding events between the aptamers immobilized on the sensor surface and their target molecule: virus proteins or intact viruses according to different SPR configurations. The methodological quality of each study was assessed using QUADAS-2, and a meta-analysis was conducted with the CochReview Manager (RevMan) Edition7.12.0 Data were analyzed, focusing on the types of viruses, the virus target, and the reference method. The pooled sensitivity was 1.89 (95%, CI 1.29, 2.78, I^2^ = 49%). The analysis of different types of plasmonic sensors showed the best diagnostic results with the least heterogeneity for SPR conventional configurations: 3.23 (95% CI [1.80, 5.79]; I^2^ = 0%, *p* = 0.65). These findings show that even though plasmonic biosensors effectively analyze viruses through aptamer approaches, there are still big challenges to using them regularly for diagnostics. Practical considerations for measuring label-free interactions revealed functional capabilities, technological boundaries, and future outlooks of SPR virus aptasensing.

## 1. Introduction

Rapid tests at the point of patient care could be highly beneficial due to the immediacy of results. They can be used outside healthcare settings to detect infectious agents causing viral diseases [1]. The advantages of these tests concerning speed, reliability, selectivity, and sensitivity should be considered to establish the most appropriate screening and diagnostic strategies for virus control [2,3]. Therefore, the acceptability, sensitivity, and cost of each type of test, the availability of resources, or the need to provide results concerning transport and in vitro diagnostic testing must be addressed to enable their implementation outside hospital settings [4].

In this context, aptasensors emerge as new diagnostic methods, thanks to advances in biotechnology and nanotechnology for detecting virus-caused diseases [5,6,7]. These promising analytical devices combine a biological recognition element called an aptamer, which can recognize and react with the target molecule (analyte), and a transducer system (optical, electrochemical, piezoelectric, or magnetic) that generates a signal proportional to the analyte concentration interacting with the aptamer [8,9,10,11,12,13,14,15]. Aptamers are small, single-stranded DNA or RNA sequences (10–100 nucleotides) that fold into well-defined, stable, three-dimensional structures [16,17,18,19,20]. This feature allows them to interact efficiently with miscellaneous molecules, from metal ions and small organic compounds like amino acids or nucleotides to large molecules like proteins, viruses, bacteria, or cells [17,18,20,21,22]. Due to their specificity, aptamers are often compared to antibodies [17,23,24,25]. Aptamers’ generation by in vitro selection methods may be more cost-effective than antibody production and purification. Additionally, aptamers can be chemically modified or integrated into nucleic acid nanostructures without affecting the affinity [26]. Unlike antibodies, the denaturation of aptamers under unfavorable conditions is reversible, allowing them to be incorporated into different detection platforms and reused without losing functionality [16,18,20].

Conventional virus detection methods can be limited by technical complexity, cost, and false positives or negatives [18,27]. Aptamer-based assays could mitigate these drawbacks by enabling the precise measurement and effective identification of viruses in complex matrices. Due to their adaptable selection, compact size, exceptional stability, and superior sensitivity, aptamers are good candidates to surpass antibodies as the primary recognition molecules in virus diagnostics. Currently, several aptasensor-based strategies for virus detection have been described, such as surface-enhanced Raman spectroscopy (SERS) [28,29]; surface plasmon resonance, SPR [30,31]; fluorescence [32]; colorimetry [33]; and electrochemistry [14]. SPR aptasensors detect interactions at the interface between the surface where the aptamer is located and the solution containing the target molecule by measuring changes in the refractive index [34]. This allows direct, real-time measurements without the need for labeling or sample purification.

**Figure 1:**
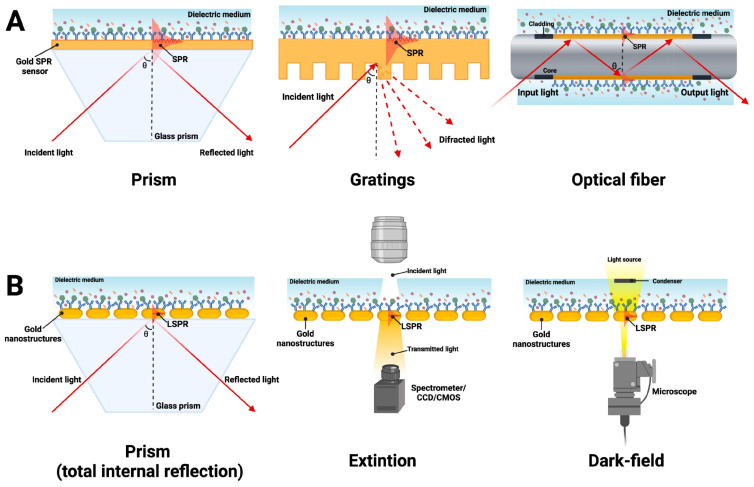
Most common SPR configurations using (**A**) an SPR in a prism-coupling configuration, grating-based couple, and optical fiber, respectively, and (**B**) an LSPR through total internal reflection measurement, extinction measurement, and dark-field measurement by a microscope. Adapted with permission from Ramirez-Priego et al. [35] Copyright © (2024) Elsevier.

The principle of SPR aptasensors is shown in Figure 1. In a typical SPR aptasensor, the aptamer is immobilized on a metal surface, usually gold. The binding between the analyte (virus or its proteins) and the aptamers changes the thickness of the gold surface and, consequently, the refractive index. The analyte bound on the surface can be quantified by measuring the angles or intensity of polarized light, which is detected by the optical transducer and converted into detectable signals [34,35]. The SPR signal is generally proportional to the molecular weight and refractive index increase and is expected to be positive and increase with the number of detected molecules [36]. The intimate contact between the aptamer and the transducer system determines these devices’ exceptional sensitivity and specificity.

SPR aptasensors have several advantages over other devices, such as miniaturization and automation [37]. Additionally, the detection approach is faster than other SPR detection modes, requiring fewer steps and less time. Due to space constraints, most small molecules can bind to aptamers only with a one-site binding configuration. However, the main advantage of SPR aptasensors in detecting viral infections is their ability to detect recombinant membrane antigens directly, and the whole virus using sandwich detection formats [22].

Previous works have focused on SPR platforms for detecting viruses using aptamers as biorecognition elements [37,38]. However, their feasibility according to the combined effect of the aptamer design, the type of virus, and the analytical performance has not been sufficiently addressed from a holistic and systematic perspective. Therefore, this review aims to identify recent trends in SPR aptamer-based biosensing of clinically relevant viruses using a methodology based on the meta-analysis of systematically obtained data. This work particularly concentrates on the capacity of SPR aptasensors to develop diagnostic tests that are ready to be used at the point of care.

## 2. Materials and Methods

### 2.1. Search Strategy and Information Sources

This systematic review was conducted in strict accordance with the Preferred Reporting Items for Systematic Reviews and Meta-Analyses (PRISMA) guidelines [39]. The comprehensive literature search encompassed several key scientific databases, including Web of Science, SCOPUS, and PubMed, and was focused on publications dated from 1 January 2014, to 1 December 2024. To ensure a thorough investigation, the keywords utilized for the search were “aptamer”, “virus”, and “plasmon”, strategically combined using the Boolean operators “AND” and “OR” to capture a wide range of relevant studies. Each database was queried without applying any filters, which maximized the retrieval of potentially pertinent articles. Subsequently, the literature obtained from this search was meticulously screened using the PRISMA checklist, allowing for a structured and systematic evaluation of the identified studies. The literature search was conducted from the main databases, including Web of Science, SCOPUS, and PubMed, considering publication dates from 1 January 2014, until 1 December 2024. The keywords used for all three databases without any filters were “aptamer”, “virus”, and “plasmon”, combined using the Boolean operators “AND” and “OR”. The retrieved literature was manually screened using the PRISMA checklist.

### 2.2. Eligibility Criteria and Selection Process

The inclusion criteria for this review were carefully defined to ensure a focused and relevant selection of studies. Specifically, the criteria encompassed the following: (i) original research articles that employ plasmonic biosensing technology combined with aptamers for the diagnosis of viral infections, highlighting the innovative use of these methods in clinical settings; (ii) studies that thoroughly evaluate the performance metrics and binding characteristics of aptamers that specifically target either viral proteins or intact viruses, providing insights into their diagnostic potential; and (iii) a variety of diagnostic test assays, including both cross-sectional studies and case–control studies, which contribute valuable data on the effectiveness of these diagnostic approaches.

Several types of publications were excluded to maintain the integrity of the review. These exclusions included studies published as book chapters, narrative or systematic reviews, conference proceedings, dissertations, letters to the editor, and short communications, as well as articles that did not present appropriate empirical data or were published in languages other than English.

The search results were systematically imported into the Zotero reference manager, where we ensured an efficient organization of the references. Duplicates were meticulously identified and removed to avoid redundancy in our analysis. Following this, the remaining articles were rigorously screened against the established eligibility criteria, focusing on their titles and abstracts to determine relevance. The abstracts deemed pertinent were then subjected to comprehensive full-text screening, allowing us to summarize and analyze the key findings and contributions of each study to the field of viral infection diagnostics.

### 2.3. Study Risk of Bias Assessment

The articles extracted from the PubMed, Scopus, and Web of Science databases were meticulously evaluated for quality using the Quality Assessment of Diagnostic Accuracy Studies 2 (QUADAS 2) framework. This assessment followed the detailed guidelines outlined in the Cochrane Handbook for Reviews of Diagnostic Test Accuracy [40]. For each retrieved article, the author systematically addressed a set of predefined questions, recording the responses—“no”, “yes”, or “no information”—in a comprehensive Excel table for clarity and organization. The QUADAS 2 tool encompasses 11 specific questions that are divided into four critical domains: patient selection, index test, reference standard, and flow and timing. Each question is designed to provide insight into different aspects of the study’s methodology and validity. Based on the responses given for each domain, the risk of bias was classified as “low”, “high”, or “no information”, allowing for a nuanced understanding of the potential limitations within each study. To ensure a thorough analysis, all findings were graphically represented using the Cochrane RevMan software, facilitating easier interpretation and comparison of the data.

### 2.4. Data Collection Process

A comprehensive review was carried out on a selection of publications that adhered to specific inclusion criteria for data collection and extraction. The articles deemed eligible were systematically categorized according to the type of virus they investigated and the associated plasmonic platforms utilized, thereby providing an organized and insightful overview of the current landscape in this rapidly evolving field.

From the selected articles, a range of detailed data points were extracted for descriptive analysis and comparative evaluation. These included the specific types of viruses studied, the various refinements made to the aptamers used for detection, the origins of these aptamers (including whether they were derived from natural sources or synthesized in the lab), and the target biomarkers that were identified for diagnostic purposes. Additionally, the review encompassed the clinical applications of the identified techniques, the types of biological samples utilized in the studies, the assays employed for testing, as well as the diagnostic techniques and corresponding treatments outlined, including the specific routes of administration for these therapies.

### 2.5. Statistical Analysis

The meta-analysis was conducted using the Cochrane RevMan software, which also facilitated the generation of a comprehensive summary of methodological quality in accordance with the QUADAS-2 bias assessment framework. To visualize heterogeneity among the studies, forest plots were generated. In cases where heterogeneity could not be adequately addressed, random effects models were utilized to account for the variability in study results. I^2^ tests were employed to measure the degree of heterogeneity, with values greater than 50% indicating statistically significant variations among the studies.

Subgroup analyses were systematically performed, taking into consideration various characteristics of the selected studies, such as the type of virus being investigated, the configuration of the plasmonic sensors used, the nature of the clinical samples analyzed, the specific viral targets, and the reference methods employed, as well as the affinity constants and sensitivity metrics observed in the studies. To evaluate the influence of smaller studies on the overall findings, funnel plots were constructed. Throughout all statistical analyses, a *p*-value of less than 0.05 was deemed statistically significant, ensuring the robustness and reliability of the results obtained.

## 3. Results

### 3.1. Retrieved Studies

**Figure 2:**
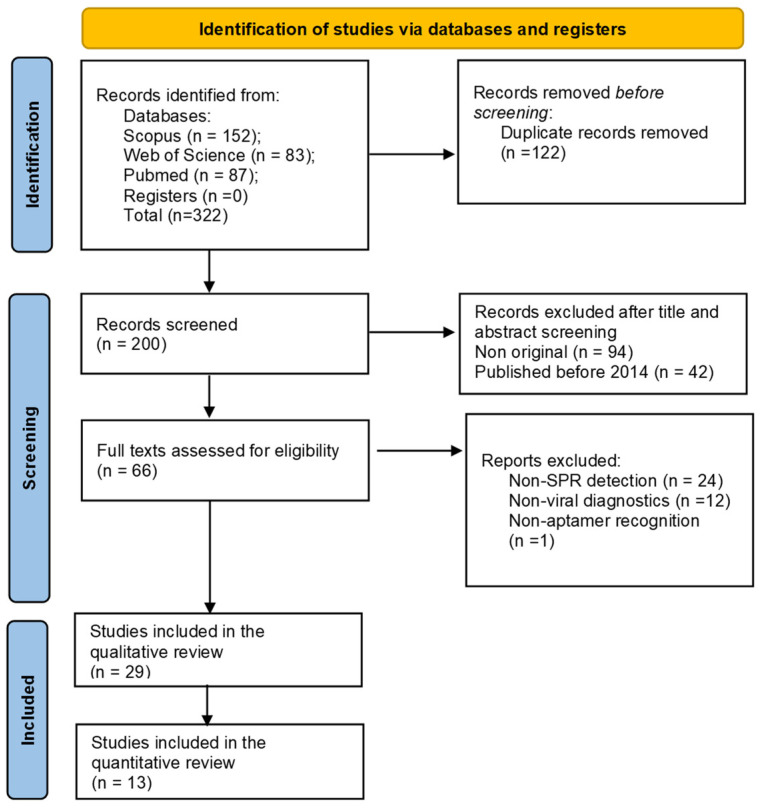
PRISMA 2020 flow diagram for article selection in this systematic review.

The number of studies identified in the three databases was 322 (Figure 2). Before screening, 122 duplicates were removed. Of the remaining 200 articles, 66 were appraised for full-text assessment once the eligibility criteria were considered. Finally, 28 studies met all of the inclusion criteria and were selected for qualitative and quantitative review. The analysis of these articles allowed the extraction of data related to SPR-based aptasensors focused on virus detection for diagnostic purposes.

Table 1 displays the key characteristics of the included studies according to the type of aptamer, target virus, plasmonic technology, detection format, and limit of quantification.

### 3.2. Quality Assessment

More than half of the studies had a low risk of bias assessment. All of the selected articles for the quantitative analysis took advantage of biological fluids to test the diagnostic potential of the aptasensors, using either real, artificial, or spiked clinical samples. However, only 15% of the studies displayed a low-risk of bias in the patient selection domain. This result is because almost fifty percent of studies focused on the characterization of aptamers instead of assessing the feasibility of the analytical tool. For this reason, the quantitative analysis was only performed in those articles that displayed the sensitivity of the plasmonic device. Most articles investigated the test accuracy by comparing the results with another diagnostic technique. In this sense, almost all of the articles within the quantitative analysis reported a cut-off or a limit of detection value. Only one article described the results solely in negative samples. As for the flow and timing domain, most articles did not explain in detail whether the index and reference tests were performed during the same time or were characterized before storage. Finally, all of the articles showed appropriate experimental designs and stated the research aims.

### 3.3. Model of Virus Target

Among the 29 selected articles, nine studies developed plasmonic aptasensors to detect hemagglutinin proteins or the influenza virus. Ten studies focused on developing aptamers to determine SARS-CoV2, while five determined the human immunodeficiency virus (HIV) using SPR biosensors. The rest of the retrieved articles studied aptamers against the Crimean Congo Hemorrhagic Fever virus (one), diarrhea virus (one), norovirus (one), dengue virus (one), and Mpox virus (one) through plasmonic platforms.

Most studies aimed to detect virus proteins [41,42,43,44,47,48,49,50,51,52,54,55,57,58,59,60,61,62,63,64,65,66,69], whereas four studies [45,46,67,68] focused on whole virus detection, and two determined protein genes [53] or pseudo viral particles [56]. Other biomolecules like enzymes were also used as virus targets. For instance, all of the selected articles provided HIV characterization using plasmonic aptasensors concentrated on examining the activity of anti-reverse transcriptase enzymes [60,61,62], evaluating the interaction with envelope glycoproteins, Gp120 and Gp41 [64], and detecting the tat protein [63]. Similarly, SARS-CoV-2 SPR-based aptasensing addressed the determination of SARS-CoV-2-related proteins such as the nucleocapsid (N) protein [50,52,53,59], the spike (S) protein [54,55,57,58], and the interaction of an aptamer directed against SARS-CoV-2SARS-CoV-2 RNA [51]. In contrast, three articles reporting the diagnosis of the influenza virus detected the whole H5N virus [45,46]. The other influenza virus-related studies dealt with various hemagglutinin subtypes [41,42,44,47,48,49] and the influenza virus nucleoprotein [43]. Other studies reported the interaction of the Crimean Congo fever virus nucleoprotein [65], the norovirus capsid protein [66], or the Mpox A29 protein [69] to their corresponding selected aptamers. Lastly, two whole virus strategies described dengue and diarrhea virus approaches [67,68].

### 3.4. Type and Origin of Aptamers

Twenty-five selected articles utilized DNA aptamers to analyze virus proteins or whole virus particles. The remaining studies described detection approaches based on RNA aptamers [47,48,63].

Ten studies reported the identification of aptamers using either library synthesis [48] or the selection amplification process known as the systematic evolution of ligands by exponential enrichment (SELEX) [43,45,46,47,49,50,63,65,67]. Conventional SELEX appeared in five articles [49,50,63,65,67]; three studies employed graphene oxide-based SELEX [45,46] and magnetic SELEX [43]; and another used the counter-SELEX method [47].

The rest of the studies involved previously selected aptamers, which included various modifications before analysis [42,44,51,52,53,54,60,62,64,66,67,68]. For instance, four studies displayed aptamers with 3D structures based on the design of G-quadruplexes [64], a three-way junction [44], or T-shaped aptamer structures [52]. Four studies comprised chemical modification through gold nanoparticles [66,67,68] and quantum dots [53] in four studies. Other modification strategies included the biotinylation [42,54,65] and thiolation of aptamers to provide stable immobilization surfaces [51].

### 3.5. Sensing Detection Schemes and Assay Formats

Most sensing schemes reported conventional SPR configurations, although two articles included structural modifications to enhance detection [55,63]. Specifically, two studies described working strategies based on fiber optic SPR sensing [50,52], and another employed SPReTIRE detection [63].

Four approaches described LSPR-based developments involving SARS-CoV2 [52,54,69], dengue [68], and influenza virus detection [44]. Additionally, one of them reported LSPR coupled to fiber optic nanoprobes [52] and another, an integrated colorimetric detection system [68] that detected whole virus organisms [52,68].

Regarding the assay format, nineteen articles referred only to the kinetic analysis of daptamers with their target virus proteins by SPR-based methods [41,42,43,47,48,49,50,52,55,56,57,58,59,60,61,62,65,66]. The other studies provided the direct detection of whole viruses (four), RNA (one), and virus proteins (eight). The dissociation constants were within the nanomolar range (0.49–69.06 × 10^−9^ M) in most cases.

The immobilization of aptamers was the most common strategy to examine either the binding affinity or the detection of the analyte (all but four). As an amplification of this format, various studies employed sandwich-based assays comprising a dual aptamer detecting scheme to recognize the whole influenza virus [46], the nucleocapsid or S protein of the SARS-CoV-2 virus [52,57], norovirus [66], and Mpox virus [56]. Contrarily, aptamers were used as analytes in four studies that determined HIV behavior against infection in HEK293 cells [60,61,62] and another that comprised the binding affinity of the Crimean fever virus [65]. SPR technology has been commonly applied to directed affinity binding analyses between target proteins and aptamers. In this context, these studies took advantage of immobilizing the target virus protein to investigate the kinetics of the protein to the aptamer using a 1:1 ratio. This approach could be advantageous for evaluating specific interactions between target proteins, viruses, and aptamers, either as a preliminary step in the analysis or as a method to enhance the aptasensors signal.

Additionally, several approaches comprising immobilized aptamers used functionalized surfaces such as the biotin–streptavidin/neutravidin method [43,45,46,47,48,65] and nanoparticle [46,50,51,52,66,67,68] or quantum dot structures [53].

### 3.6. Analytical Outcomes and Reference Methods

All of the studies except eight [42,43,47,48,49,60,61,62,64] evaluated the assay sensitivity. The limit of detection (LOD) in a molar ratio [37,44,52,54,55,63,66] or EID_50_/mL (TCID_50_/mL) [45,46,67] was the selected method to determine sensitivity.

The influenza virus detection was mainly focused on the affinity of aptamers instead of the sensitivity analysis. Six studies aimed to determine the affinity binding constants between influenza-related proteins (recombinant hemagglutinins and influenza nucleoprotein) and complementary aptamers [41,42,43,47,48,49]. Among the other influenza aptasensors, two provided sensitivity values based on the whole virus detection of 200–2.09 × 10^5^ EID_50_/mL or viral particles [45,46], while another reported hemagglutinin detection between 1 and 100 pM levels [44].

Regarding SARS-CoV2 aptasensors, most studies focused on the detection limits expressed in molar concentration [50,52,53,54,55]. Two methods showed detection limits within the nanomolar range for the S1 glycoprotein (0.26–37 nM) [54,55], and the other two performed nucleocapsid protein analyses in the picomolar [52] and nanomolar concentrations [50]. The remaining SARS-CoV2-related articles tested the aptamer recognition of RNA-positive samples using colorimetric enhancement and N-gene detection at picomolar levels.

HIV SPR-based aptasensing only included sensitivity outcomes in LOD levels in one study to detect the Tat protein at 1 pM levels [63]. The other HIV aptasensors concentrated on the cytotoxicity testing on HEK293 cells and the inhibition of pseudo-HIV particle infection in HEK293 cells [60,61,62]. The stability of G-quadruplex aptamers in human serum concerning the recognition of HIV glycoproteins 120 and 41 was also reported [64].

Concerning other virus aptasensors, measurements of whole viruses through TCID_50_/mL units and graphic detection were reported for diarrhea [67] and dengue [68] viruses, respectively. Although the number of Crimean fever virus copies was determined using ELISA in another article, SPR feasibility was also applied to examine the affinity binding. Lastly, the lowest LOD emerged when evaluating the norovirus capsid protein at attomolar levels in buffer samples (70 aM).

The specificity was also examined to determine the analytical performance of SPR aptasensors. Most of the studies (71%) considered the assessment of the aptamer selectivity using non-specific viruses or proteins and antibodies such as bovine serum albumin, human serum albumin, and immunoglobulins.

As for the use of biological samples, different types of fluids, including chicken serum [44], duck feces [45], nasopharyngeal samples [50,51], cold chain foods [52], seafood seawater [53], artificial saliva and serum albumin [54], and human serum [53,64,66] were tested to demonstrate the clinical applicability of SPR aptasensors.

Several methods considered the inclusion of another method as a reference. The range of reference methods varies according to the target virus and assay format including ELISA [47,66], ELASA [65], and ELAA [42,49], PCR [67], UV–vis spectrum [68], ellipsometry [63,64], isothermal titration calorimetry [60], NMR [61,62], AFM [55], colorimetric determination [51], and circular dichroism spectrum analysis [45,46]. Similarly, several enhancement procedures involving fluorescence dyes [44], colorimetric assays [67], quantum dots [46,53], fiber optics [50,52,55], or ellipsometry [63,64] were used. These methods were selected based on the specific biomolecular characteristics of the target analyte. For instance, both ELISA and SPR demonstrated comparable results, consistently yielding nanomolar Kd values across various experimental conditions. Likewise, SPR aptasensors demonstrated comparable sensitivity values with either immunoanalytical techniques or genomic sequencing.

### 3.7. Sensitivity and Subgroup Analysis

**Figure 3:**
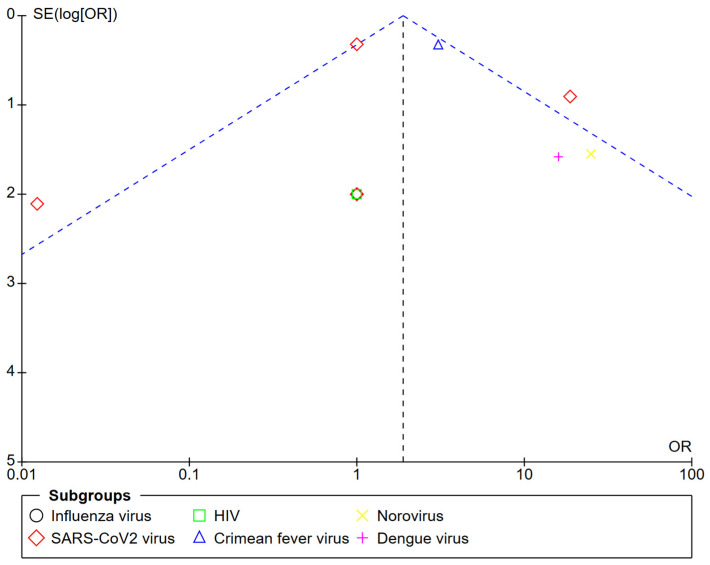
Funnel plot of the diagnostic odds ratio according to the types of viruses.

The symmetric distribution of the studies subjected to the quantitative analysis according to the number of positive and negative samples in each study is presented as funnel plots (Figure 3). The effect of the plasmonic configuration, the type of virus and target, the kinetics, the biological fluid, and the use of a reference method revealed the same symmetric distribution.

The meta-analysis also examined the impact of these variables by considering the sensitivity analysis and the heterogeneity between the selected studies. First, the sensitivity analysis provided a better understanding of the potential limitations and the degree of uncertainty surrounding the results according to the negative and positive samples utilized in each study. Secondly, the differences in the measured outcomes found across the studies provided information about the heterogeneity of the overall findings, thus offering the quality and usefulness of evidence-based practices.

**Figure 4:**
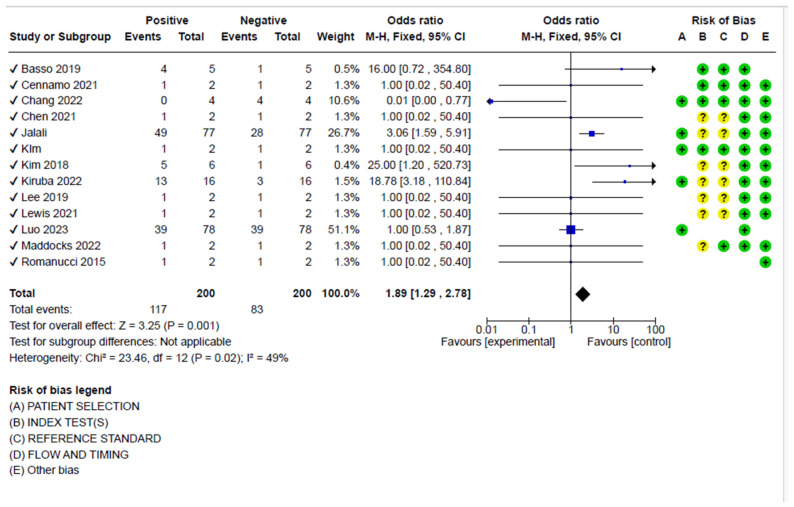
Analysis for pooled diagnostic odds, including the risk of bias for the articles selected for the meta-analysis. Basso 2019 [68]; Cenammo 2021 [55]; Chang 2022 [50]; Chen 2021 [53]; Jalali [65]; Kim [45]; Kim 2018 [66]; Kiruba 2022 [51]; Lee 2019 [44]; Lewis 2021 [54]; Luo 2023 [52]; Maddocks 2022 [41]; Romanucci 2015 [64].

The sensitivity analysis considering the positive and negative samples showed a pooled diagnostic odds ratio of 1.89 (95%, CI 1.29–2.78), showing moderate heterogeneity (I^2^ = 49%, *p* < 0.001) (Figure 4).

The sensitivity analysis based on the different types of viruses showed that the differences between subgroups were not statistically significant, with a *p*-value of 0.09 and a heterogeneity value (I^2^) of 48.2% (Figure S1A). The examination of the influenza virus articles indicated their similarity (*p* = 1.00, I^2^ = 0%). In contrast, the analysis of SARS-CoV-2 articles showed the highest heterogeneity, with a *p*-value of 0.01 and an I^2^ value of 65%. When considering whether the target was the whole virus or a specific protein, only two studies focused on the whole virus, with a heterogeneity value of *p* = 0.28 and I^2^ = 16% (Figure S1B). Regarding the type of SPR sensors, the sensitivity analysis yielded a diagnostic odds ratio of 1.66, indicating that the results were somewhat consistent (CI: 1.20 to 2.29) (Figure S1C). This analysis showed moderate heterogeneity (*p* = 0.009, I^2^ = 53%). Specific approaches, like surface plasmon resonance (SPR), showed no significant differences in heterogeneity (*p* = 0.65, I^2^ = 0%). Localized surface plasmon resonance (LSPR) biosensors had a *p*-value of 0.24 and I^2^ = 28%, while SPR fiber optic devices had *p* = 0.12 and I^2^ = 54%. The subgroup analysis focused on the kinetics measurements did reveal statistically significant differences between studies that measured dissociation constants and those that did not examine affinity (*p* = 0.008, I^2^ = 85.9%). In contrast, the studies that did not measure affinity showed no heterogeneity (I^2^ = 0%) (Figure S1D). Lastly, when comparing studies using a reference method, there was again no heterogeneity (*p* = 0.59, I^2^ = 0%). However, the differences within studies utilizing a reference method were not statistically significant (*p* = 0.09, I^2^ = 45%) compared to studies that did not use a reference method, with a *p*-value of 0.03 and I^2^ = 61% (Figure S1E).

## 4. Discussion

This systematic review and meta-analysis aimed to determine the reliability of SPR aptasensors for detecting virus-related interactions while examining recent trends to enhance their performance. The data search identified 29 articles built on SPR biosensors that utilized aptamers as analytes or recognition elements to monitor virus interactions.

The selected SPR-based virus aptasensors took advantage of the sensitivity provided by the plasmonic phenomenon when measuring the refractive index changes that occur due to the interaction of aptamers with protein or whole virus molecules. The different configurations that adopt aptameric SPR biosensors depend on the sensing mechanism, the virus target, or the immobilization strategy.

In this sense, SPR aptasensors based on conventional configurations still provide the most abundant strategies to detect the target virus. This circumstance is directly tied to the extended use of SPR biosensors to investigate the dynamics of aptamers’ interactions with their target virus components. For this reason, basic SPR assays usually exploit a direct detection approach consisting of aptamer immobilizations on the sensing surface followed by the recognition of the virus protein using only one-site binding. The direct detection strategy was present in almost all selected articles. Nevertheless, several approaches have benefitted from the streptavidin–biotin interaction to immobilize a biotin-modified aptamer [43,45,46,48,65]; others make use of signal amplification tags such as nanoparticles [46,50,51,52,66,67,68] or quantum dot structures [53]. As an extension of this format, three distinct studies utilized sandwich assays incorporating a dual aptamer detection system to detect either whole or protein viruses [46,52,57,67,69]. Therefore, direct virus detection formats are the first choice for encouraging outcomes concerning sensitivity, specificity, and cost-effectiveness since only four studies did not immobilize aptamers on chip surfaces to monitor virus interactions. This outcome coincides with the lack of heterogeneity observed when evaluating the distribution of positive and negative samples in each study showing the best diagnostic odds ratio.

Although the fundamental sensing mechanism was SPR, some approaches used variations of this detection mode, such as LSPR, resonance-enhanced total internal reflection ellipsometry (SPReTIRE), or fiber optic transducers. LSPR configurations, compared to angle-resolved arrangements, allowed more straightforward and stable measurements without the need for prisms or polarizers [44,52,54,56]. Moreover, LSPR biosensors can enhance the spatial resolution of plasmonic platforms by utilizing plasmonic nanomaterials [56]. These distinctive characteristics enable the integration with microfluidics and, consequently, the miniaturization of LSPR aptasensors. Although the integration of microfluidics is also possible in SPR conventional formats, LSPR configurations allow the incorporation of practical microfluidic designs that can enhance the performance and efficiency of more compact systems while incorporating advanced software solutions.

Among the selected studies of this review, integrating fiber optics technology into either SPR or LSPR system configurations presented a promising advancement. For instance, an LSPR-based approach permits the direct detection of the nucleocapsid protein of the SARS-CoV2 virus using gold/silver nanoparticles functionalized with a T-shaped aptamer on Ω-shaped fiber optic surfaces [52]. The principle of FOPPR sensing technology using aptamer-functionalized sensor fibers offers an interesting approach to improving the sensitivity of SPR aptasensors to measure the affinity between the immobilized aptamer and the virus target [50,55]. However, incorporating SPR aptasensors into fiber optics necessitates a range of enhancements that facilitate the deposition of gold on the fiber core while allowing aptamer immobilization and virus detection with sufficient feasibility and minimum mechanical noise and fragility.

The SPR imaging configuration is solely represented by a unique approach using a specific aptamer that recognizes the S protein of SARS-CoV2 [57]. This strategy reported the aptamer kinetics but did not consider the diagnostic assay assessment.

Merging the surface plasmon resonance phenomena with enhanced total internal reflection ellipsometry (SPRe-TIRE) demonstrated an interesting aptasensing arrangement. This singular approach has been successfully applied to detect HIV [63], resulting in improved sensitivity as SPR waves travel across the metal surface. Another unusual SPR strategy involves an SPR colorimetric-based assay that exhibited its diagnostic value using 16 clinical samples [51]. However, this strategy was based on the SPR-related absorbance measured as the wavelength shift changed in aggregated gold nanoparticles instead of exploiting the SPR phenomenon.

The studies reviewed encompassed diverse virus types, ranging from Influenza A to SARS-CoV2 and, more recently, to the Mpox virus. The analysis of the group differences between the investigated viruses showed moderate heterogeneity, thus indicating low variations in their diagnostic accuracy according to the number of positive and negative samples. Because the most employed assay format was based on aptamer immobilization, the predominantly recognized molecule was a viral antigen. The aptamers commonly screened by the SELEX method had corresponding target proteins including envelope, capsid, nucleoprotein, and functional proteins [27]. Envelope proteins, such as hemagglutinin (HA) and SARS-CoV-2 spike glycoproteins, provide external sites to recognize aptamers and are the preferred option for SPR aptasensors, as confirmed in this review. Secondly, capsid and nucleocapsid proteins constitute a solid alternative for viral diagnosis, as demonstrated in norovirus and SARS-CoV2 N-protein SPR-based aptamer applications despite being assembled to package the viral nucleic acid.

The sensitivity of aptasensors is a critical factor that significantly influences their effectiveness in detecting the wide range of viruses herein described. Enhancing sensitivity using advanced aptasensor designs that involve conformational changes upon target binding is a suitable approach to reach this aim. Signal amplification strategies also play a vital role in addressing sensitivity challenges. In addition to target-induced dissociation, competitive displacement has contributed to higher accuracy and specificity, making it possible to identify virus targets even in minimal concentrations. Similarly, specificity is essential when developing aptasensors, as it directly influences the accuracy and reliability of detection. High specificity ensures that the SPR aptasensor only interacts with the intended target virus, minimizing the risk of false positives that arise from cross-reactivity with non-target molecules. This is particularly important in clinical applications, where the presence of similar compounds could lead to misleading results. Enhancing specificity often involves understanding the biorecognition mechanisms and optimizing intermolecular interactions within the aptamer. Elucidating the binding domains and making precise sequence changes can significantly improve an aptamer’s affinity and selectivity for its virus target. However, several studies still lack appropriate determinations of either sensitivity or specificity values, generating a gap between SPR lab approaches and onset clinical applications.

As for the performance comparison, no group differences were observed between the studies with and without reference methods, although, the heterogeneity among the approaches that used a reference method was slightly lower. Despite these outcomes, validating the results with a reference method is crucial for assessing the diagnostic capability of an analytical technique. Immunochemistry methods (ELISA and ELAA), fluorescence, nanomechanical, voltammetry, and CRISPR were the methods compared to SPR aptamer arrangements. The similarity between SPR aptasensors and reference methods highlights the reliability and accuracy of SPR aptasensors in measuring binding affinities. Such comparability reinforces the confidence in adopting SPR technologies as reliable alternatives or complements to traditional standard methods.

The intricate complexity inherent in biological samples is crucial in driving feasible aptamer applications. In this review, most studies have concentrated on serum as the primary biofluid for virus identification. Because serum presents exceptional complexity, many studies rely on diluted samples. Moreover, alternative biological fluids such as nasopharyngeal, artificial saliva, and even duck feces offer viable solutions in virus diagnosis to overcome the requirements of diagnosis test assays. Nevertheless, utilizing buffers instead of biological samples remains the typical method, thus indicating the failure of many studies to replicate clinical conditions. These findings highlight a significant shortcoming in many studies: the difficulty of exporting SPR aptasensing to applications outside laboratory settings. Specifically, the presence and distinct characteristics of foulants—such as proteins, lipids, and other biomolecules—found in biofluids can profoundly disrupt the analytical signal generated by the aptasensor. This disruption can lead to a deterioration in both assay sensitivity and specificity. As a result, sensing strategies must extend beyond merely developing robust antifouling materials to enhance the durability and stability of immobilized aptamers. These strategies should also include the integration of advanced separators within microfluidic systems. Despite the promising advancements in SPR technology, there remains a substantial need for further research and refinement to achieve optimal performance in real-world applications. Therefore, prioritizing the optimization process is essential for enhancing the effectiveness and accuracy of SPR-based virus detection using aptamer approaches.

This review points out key limitations regarding the selected studies. First, some studies did not provide information on the test assay accuracy, thus limiting the quantitative analysis to half of the collected documents. Furthermore, the sample sizes employed in these studies may have limited statistical analysis. On the other hand, many studies failed to report true positives, false positives, true negatives, and false negatives, which affects the heterogeneity of the meta-analysis due to varying positivity thresholds. While diagnostic performance was presented as odds ratios, we could not associate sensitivity and specificity with the area under the ROC curve because of a lack of data on false results. Consequently, current research on the applications of SPR aptasensors in virus analysis still lacks independent index test sets, which are necessary for generalizing diagnostic accuracy, as indicated by the studies included in this review.

## 5. Conclusions

This systematic review explores the significant advancements and challenges of SPR aptasensors as diagnostic tools for virus detection. Despite achieving ultrasensitive and reliable analytical performance, their use to diagnose viral infections occasionally remains limited to determining affinity interactions. Other obstacles like nonspecific protein adhesion in complex biological media persist, complicating the development of reliable diagnostic devices in clinical scenarios. Key considerations for advancing plasmonic aptasensors include normalizing the integration of clinical samples, determining the minimum sample size for statistically significant results, and estimating diagnostic accuracy using positive or negative outcomes. To secure their place as reliable diagnostic methods, it is crucial to create stable biological surfaces that maintain affinity and specificity toward target viruses.

However, the selectivity of aptamers encompasses a range of techniques, such as chemical alterations of the nucleotide sequences to improve functionality, the strategic insertion of unnatural nucleotides to increase diversity and specificity, and the capping of aptamer ends to protect against enzymatic degradation. Thus, the SELEX (systematic evolution of ligands by exponential enrichment) procedure is crucial in augmenting aptamer binding affinity. Achieving a specific interaction not only boosts the performance of the aptasensor but also contributes to better dynamic range and lower detection limits. Therefore, addressing sensitivity issues in SPR aptasensor technology is fundamental to improving biosensing platforms, opening new possibilities for real-world applications and improving outcomes across health and environmental sectors.

Using innovative long-term stability strategies is essential for ensuring an effective coverage of immobilized aptamers, thus enhancing the bioactivity of sensing areas and preventing the adhesion of undesired biomolecules.

## Figures and Tables

**Table 1:** Characteristics of the selected studies based on different types of viruses according to the target virus, the assay format and aptamer design, instrument configuration, kinetics, limit of detection, and biological sample.

Type of Virus	Target Virus	Aptamer Design	Assay Format	Instrument Configuration/Reference Method	Kinetics/Specificity	Limit of Detection (LOD)/Biological Sample
Influenza	Hemagglutinin (HA) protein	DNA aptamer derivatized with a thiol (SH-C6-) and a methylene blue modification	Affinity binding and detection	SPR/square wave voltammetry	K_D_ = 1.79 × 10^−8^ M	LOD = 10 nM HA/artificial saliva [41]
	Recombinant HAs: H1, H3, H5, H7, and H9	DNA aptamers and biotinylated derivatives G-Quadruplex	Affinity-binding HA immobilization	SPR/ELAA (with viral particles)	K_D_ = 78 nM	N/a [42]
	Influenza A nucleoprotein (infA NP)	DNA aptamers (magnetic SELEX)	Affinity binding The aptamer is immobilized using the biotin–streptavidin method	SPR as a reference method	K_D_ = 17.7 ± 3.5 nM/BSA; influenza B nucleoprotein	N/a [43]
	HA protein	Multi-functional DNA three-way junction (3WJ) tagged to an HA protein recognition aptamer	Detection: DNA3WJ aptamer immobilization	LSPR (enhancement effect by fluorescence dye)	N/a/ S protein from MERS-CoV coat protein)	LOD = 1 pM–100 nM /10-fold diluted chicken serum [44]
	Whole avian influenza virus particles of H5N2	GO-SELEX: aptamers, different target sites	Screening a cognate pair of aptamers: aptamer immobilization using the streptavidin–biotin complex method	SPR/circular dichroism (CD) spectrum analysis confocal laser scanning microscopy	N/a/ other avian influenza virus, infectious bronchitis virus, and Newcastle disease virus	LOD = 2.09 × 10^5^ EID_50_/mL in the duck’s feces (lateral flow assay strips) [45]
	H5Nx whole viruses	DNA aptamers. Multi-GO-SELEX method	Sandwich-based assay: Primary aptamer streptavidin-coated magnetic bead and biotin-labeled secondary aptamer on streptavidin-coated quantum dot AuNP	SPR/circular dichroism studies	N/a/ IF1, IF4, IF10, and IF21	200 EID_50_/mL/sample buffer [46]
	HA subtype 1	SELEX DNA aptamers HA1 subunit of subtype H1 (H1-HA1)	Biotinylated ssDNA aptamers immobilized on NeutrAvidin	SPR/ELISA	K_D_ = 64.76 ± 18.24 nM/H5-HA1 and GST proteins	N/a [47]
	Hemagglutinins (HAs): HPAI H5N1, A/H5N1/Indonesia/05/2005), and H7N7 (A/H7N7/Netherlands/219/2003	RNA library in RNA binding buffer	Competitive assay Immobilization of biotinylated tetravalent glycan with different concentrations of aptamer	SPR	K_D_ = 4–14 nM	N/a [48]
	H5N1, H1N1, and H3N2 subtypes of influenza A virus (subtypes of influenza A viral particles antigenically distinct)	SELEX DNA aptamers	Aptamer immobilization onto the sensor chip via a 50-biotinylated oligo	SPR/ELAA	K_D_ = 1.53–2.47 × 10^−8^ M/subtype viruses	ELAA [49]
SARS-CoV2	Nucleocapsid protein (N-protein)	DNA aptamer SELEX: NP-A48 NP-A58; NP-A61; and GNP-A15	Aptamer immobilization: ssDNA aptamer with terminal amine tagged to AuNP	fiber optic particle plasmon resonance (FOPPR)/SPR	K_D_ = 0.49–4.38 nM (SPR) K_D_ = 2.63–13.70 nM (FOPPR)/S protein; BSA	LOD = 2.8 nM (FOPPR) spiked negative samples (nasopharyngeal swabs) [50]
	SARS-CoV2 RNA colorimetric changes in the SPR peak in COVID-19	(DNA)-based aptamer	Aptamer-functionalized AuNP mixture incubated with viral RNA extracts	SPR-colorimetric-based assay	N/a/ dengue virus	SARS-CoV2 N gene Ct = 25/16 clinical samples (thirteen positive and three negative samples) [51]
	Nucleocapsid protein	T-apt@AuNPs, polyA-apt@AuNPs, and thiol-apt@AuNPs, T-shaped aptamer DNA1 containing an Np-A48 aptamer	Sandwich assay T-shaped aptamer (apt-Ag@AuNPs) for the amplification	Ω-shaped fiber optic LSPR	K_D_ = 0.024 nM, (T-apt@AuNPs)/CPN, flu A, Flu B, P1, IgG, PSA, and BSA	LOD = 9.2–28 pM/39 healthy volunteers and 39 COVID-19-infected patients and cold-chain foods [52]
	N-gene of SARS-CoV-2	SARS-CoV-2-N58 aptamer (purchased)	Thiol-modified niobium carbide MXene quantum dots anchoring N-gene-targeted aptamer	SPR	N/a/ S2-RBD protein; BSA	LOD = 4.9 pg mL^−1^/human serum, seawater, and seafood [53]
	S1 spike protein	DNA aptamer (purchased)	Immobilization of biotinylated aptamers	LSPR instrument equipped with a two-channel system	N/a	LOD = 0.26 nM, artificial saliva, and serum albumin [54]
	Spike glycoprotein	DNA aptamer specific for the recognition of the receptor-binding domain (RBD) of the SARS-CoV-2 spike glycoprotein	Aptameric sequence immobilized on a short PEG interface	SPR D-shaped plastic optical fiber (POFs)/AFM	K_D_ = 5.8 nM/BSA, AH1N1 hemagglutinin protein MERS spike protein	LOD = 37 nM/human serum (1:50 *v*/*v*) [55]
	SARS-CoV-2 SRBD or SARS-CoV-2 pseudo viral particles	DNA capturing aptamer/amino-capped aptamers	Poly (amidoamine) dendrimers conjugated to aptamer-modified chips	LSPR (nanoislands)	K_D_ = 5.8 nM (previously characterized)/SARS-CoV SRBD and Middle East Respiratory Syndrome (MERS)-CoV SRBD	LOD = 21.9 pM [56]
	S protein	DNA aptamer	DNA aptamer immobilized on gold nanoparticle-linked sandwich structure	SPRi	K_D_ = 0.82 (±0.03) nM/ 1.27 (±0.4) nM/(MERS-S), lysozyme (Lys), (BSA), and human serum albumin (HAS	LOD = 0.32 nM [57]
	COVID-19 S1 protein	Magnetic bead-assisted SELEX to discover ssDNA aptamers (five sequences)	Immobilized COVID-19 S1/binding kinetics for the S1 from wild immobilized Apt-S1-79s	SPR /CRISPR detection	K_D_ = 0.87–35.95 nM	N/a [58]
	Nucleocapsid protein	Detection of library affinity using SPR	Immobilization of the target protein binding between aptamers and NPs	SPR BIacore/Capillary electrophoresis	K_D_ = 2.18 × 10^−4^–4.21 × 10^−11^ M/SARS; MERS	N/a [59]
HIV	Anti-HIV-1 reverse transcriptase	DNA aptamers were isolated as anti-HIV-1 RT inhibitors; DNA aptamers were screened against WT HIV-1 RT in an AuNP-based colorimetric assay	Affinities of the aptamer complexes WT HIV-1 RT: DNA aptamer as analyte	SPR/isothermal titration calorimetry	K_D_ = 2.87 × 10^−6^/7.51 × 10^−8^ M	Cytotoxicity testing of DNA aptamer on HEK293T cells [60]
	K103N/Y181C double mutant HIV-1 reverse transcriptase	DNA aptamers	The binding affinity of HIV-1 RT–aptamer complexes	SPR/NMR	K_D_ = 1.56 × 10^−6^–1.46 × 10^−7^ M	KY44 could inhibit pseudo-HIV particle infection in HEK293 cells [61]
	HIV-1 RTs-RT1t49 aptamer complex	RT1t49 DNA aptamers	RT1t49 aptamer as analyte	SPR/isothermal titration calorimetry	K_D_ values of 52.8 ± 0.22 and 65.8 ± 0.52 nM	N/a [62]
	HIV-TAT(trans-activator of transcription)	RNA aptamer (already reported)	Anti-Tat aptamer immobilization	(SPReTIRE)/ellipsometry	N/a/bovine serum albumin (BSA)	LOD = 1 pM (about 1.5 pg/mL)/1.8 nM ellipsometry [63]
	HIV-1 gp120 and HIV-1 gp417	Bimolecular G-quadruplex aptamers based on Hotoda’s sequence	Binding capacity to HIV-1 gp120 and HIV-1 gp417 inhibition of HIV-1 infection in CEM cell cultures: gp120 and recombinant HIV-1(HxB2) gp417 immobilization/aptamer as analyte	SPR	The K_D_ values could not be accurately determined	Stability of G-quadruplexes in human serum [64]
Crimean Fever	Nucleoprotein (NP) CCHF virus	SELEX ss DNA aptamers use an 80-nucleotide aptamer library	Biotin immobilization of NP on a pre-coated streptavidin chip	SPR	K_D_ = 1.2 × 10^−7^–6.62 × 10^−8^ M/Dengue and Chikungunya viruses.	ELASA on 77 serum samples, including 49 positive 2.8 × 10^5^ copies/μL [65]
Norovirus	Norovirus capsid protein	Four different DNA aptamers (commercial)	Aptamer conjugated to gold nanorods immobilized onto a chip surface	SPR/ELISA	N/a (previous studies)/nonspecific control	LOD = 70 aM (buffer)/undiluted human serum samples (five positive and one negative) [66]
Diarrhea virus	Whole virus	SELEX ssDNA aptamer (cloning and sequencing steps)	Aptamer-based sandwich assay: aptamer pairs conjugated with gold nanoparticles	SPR/PCR	K_D_ = 4.08 × 10^4^,TCID_50_/mL	Sandwich with AuNP (without AuNP: 500–10,000 TCID_50_/mL) [67]
Dengue virus	Whole virus	Hairpin structure DNA aptamer (purchased) modified with SH group for binding with AuNPs	SAMN@MPA with AuNPs conjugated with immobilized aptamers	SPR/UV–visible spectrum	N/a/Zika and yellow fever viruses	Not measured, graphic for detection of dengue virus (real samples) [68]
Mpox virus	A29 protein	DNA library of the A29 protein by magnetic bead-assisted SELEX.	Immobilized A39 protein and sandwich-type binding between the aptamers and the A29	SPR/CRISPR	K_D_ = 6.8 pM and 56.4 pM/SARS-CoV-2 nucleocapsid protein (N protein) and S1-RBD, human serum albumin (HSA), and cardiac troponin I	LOD = 0.28 ng mL^−1^/human serum and saliva [69]

SPR: surface plasmon resonance; LSPR: localized surface plasmon resonance; SPReTIRE: resonance-enhanced total internal reflection; SELEX: systematic evolution of ligands by exponential enrichment; K_D_: constant of dissociation; LOD: limit of detection; TCID_50_/mL: tissue culture infectious dose 50 per milliliter; EID_50_/mL: 50 percent embryo infectious dose; AuNP: gold nanoparticles; FOPPR: fiber optic particle plasmon resonance; ELISA: enzyme-linked immunosorbent assay; ELAA: enzyme-linked aptamer assay; CRISPR: clustered regularly interspaced short palindromic repeats; NMR: nuclear magnetic resonance; AFM: atomic force microscopy; PCR: polymerase chain reaction; and BSA: bovine serum albumin.

## Supplementary Materials

The following supporting information can be downloaded at: https://www.mdpi.com/article/10.3390/bios15040245/s1, Figure S1: Subgroup analysis for pooled diagnostic odds ratio based on the type of virus (A), virus target (B), instrument configuration (C), kinetics (D) reference method (E).
