# Supplementary material for: Trends and Challenges of SPR Aptasensors in Viral Diagnostics: A Systematic Review and Meta-Analysis
Source: Biosensors (Basel). 2025 Apr 12;15(4):245. doi: 10.3390/bios15040245 (PMC12026110; doi:10.3390/bios15040245)
Supplement: Supplementary file 1 [file biosensors-15-00245-s001.zip › biosensors-3552021-supplementary.pdf]

A

Study or Subgroup	Positive Events	Positive Total	Negative Events	Negative Total	Weight	Odds ratio M-H, Fixed, 95% CI
**1.2.1 Influenza virus**						
✓ Kim 2019	1	2	1	2	1.3%	1.00 [0.02 , 50.40]
✓ Lee 2019	1	2	1	2	1.3%	1.00 [0.02 , 50.40]
✓ Maddocks 2022	1	2	1	2	1.3%	1.00 [0.02 , 50.40]
**Subtotal**		**6**		**6**	**3.9%**	**1.00 [0.10 , 9.61]**
Total events:	3		3			
Test for overall effect: Z = 0.00 (P = 1.00)						
Heterogeneity: Chi² = 0.00, df = 2 (P = 1.00); I² = 0%						
**1.2.2 SARS-CoV2 virus**						
✓ Cennamo 2021	1	2	1	2	1.3%	1.00 [0.02 , 50.40]
✓ Chang 2022	0	4	4	4	10.6%	0.01 [0.00 , 0.77]
✓ Chen 2021	1	2	1	2	1.3%	1.00 [0.02 , 50.40]
✓ Kiruba 2022	13	16	3	16	1.5%	18.78 [3.18 , 110.84]
✓ Lewis 2021	1	2	1	2	1.3%	1.00 [0.02 , 50.40]
✓ Luo 2023	39	78	39	78	51.1%	1.00 [0.53 , 1.87]
**Subtotal**		**104**		**104**	**67.1%**	**1.23 [0.73 , 2.08]**
Total events:	55		49			
Test for overall effect: Z = 0.79 (P = 0.43)						
Heterogeneity: Chi² = 14.27, df = 5 (P = 0.01); I² = 65%						
**1.2.3 HIV**						
✓ Romanucci 2015	1	2	1	2	1.3%	1.00 [0.02 , 50.40]
**Subtotal**		**2**		**2**	**1.3%**	**1.00 [0.02 , 50.40]**
Total events:	1		1			
Test for overall effect: Z = 0.00 (P = 1.00)						
Heterogeneity: Not applicable						
**1.2.4 Crimean fever virus**						
✓ Jalali 2021	49	77	28	77	26.7%	3.06 [1.59 , 5.91]
**Subtotal**		**77**		**77**	**26.7%**	**3.06 [1.59 , 5.91]**
Total events:	49		28			
Test for overall effect: Z = 3.34 (P = 0.0008)						
Heterogeneity: Not applicable						
**1.2.5 Norovirus**						
✓ Kim 2018	5	6	1	6	0.4%	25.00 [1.20 , 520.73]
**Subtotal**		**6**		**6**	**0.4%**	**25.00 [1.20 , 520.73]**
Total events:	5		1			
Test for overall effect: Z = 2.08 (P = 0.04)						
Heterogeneity: Not applicable						
**1.2.6 Dengue virus**						
✓ Basso 2019	4	5	1	5	0.5%	16.00 [0.72 , 354.80]
**Subtotal**		**5**		**5**	**0.5%**	**16.00 [0.72 , 354.80]**
Total events:	4		1			
Test for overall effect: Z = 1.75 (P = 0.08)						
Heterogeneity: Not applicable						
**Total**		**200**		**200**	**100.0%**	**1.89 [1.29 , 2.78]**
Total events:	117		83			
Test for overall effect: Z = 3.25 (P = 0.001)						
Test for subgroup differences: Chi² = 9.66, df = 5 (P = 0.09), I² = 48.2%						
Heterogeneity: Chi² = 23.46, df = 12 (P = 0.02); I² = 49%						

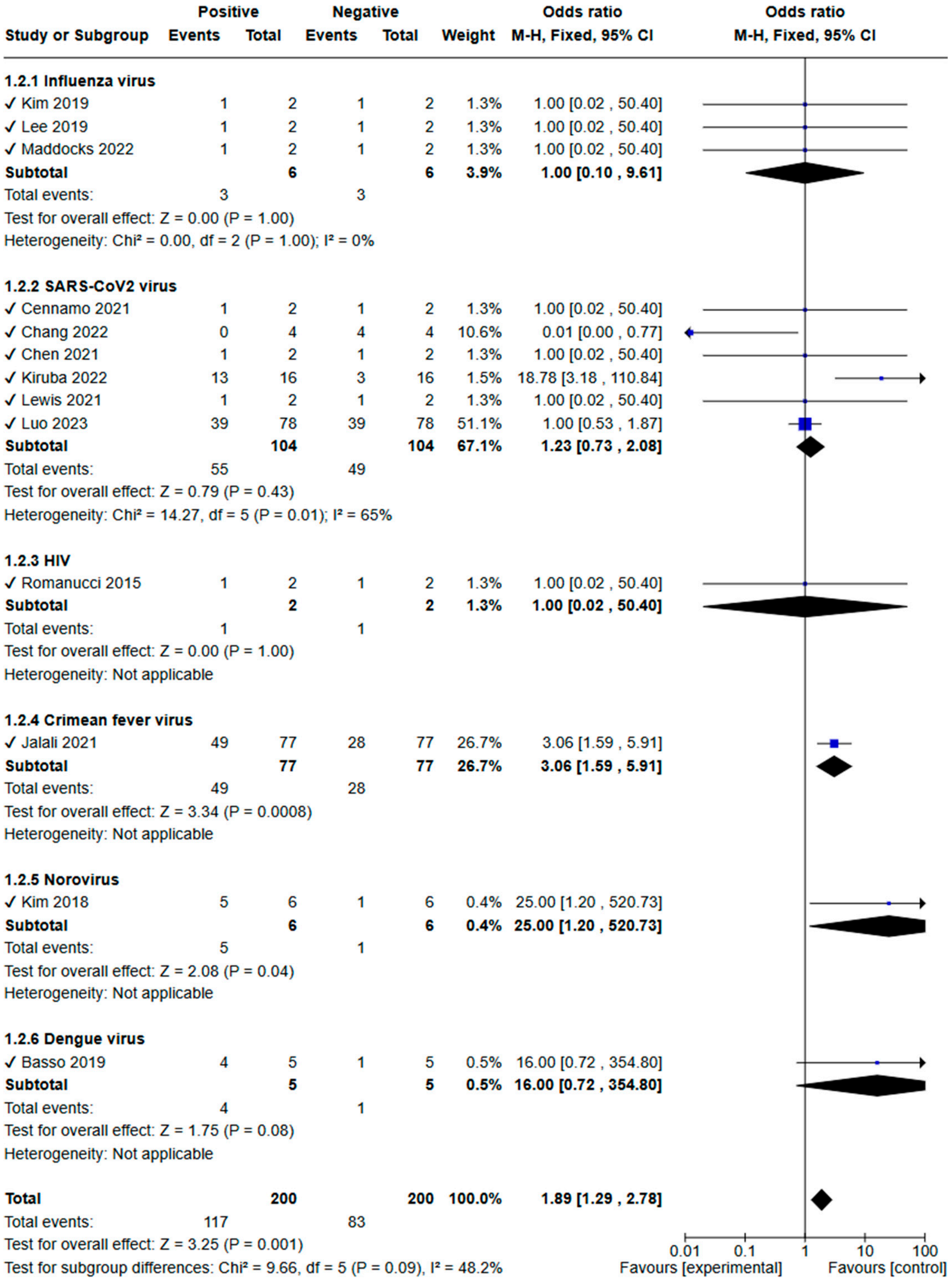

B

Study or Subgroup	Positive Events	Positive Total	Negative Events	Negative Total	Weight	Odds ratio M-H, Fixed, 95% CI
**1.6.1 Protein**						
✓ Cennamo 2021	1	2	1	2	1.3%	1.00 [0.02 , 50.40]
✓ Chang 2022	0	4	4	4	10.6%	0.01 [0.00 , 0.77]
✓ Chen 2021	1	2	1	2	1.3%	1.00 [0.02 , 50.40]
✓ Jalali 2021	49	77	28	77	26.7%	3.06 [1.59 , 5.91]
✓ Kim 2018	5	6	1	6	0.4%	25.00 [1.20 , 520.73]
✓ Kiruba 2022	13	16	3	16	1.5%	18.78 [3.18 , 110.84]
✓ Lee 2019	1	2	1	2	1.3%	1.00 [0.02 , 50.40]
✓ Lewis 2021	1	2	1	2	1.3%	1.00 [0.02 , 50.40]
✓ Luo 2023	39	78	39	78	51.1%	1.00 [0.53 , 1.87]
✓ Maddocks 2022	1	2	1	2	1.3%	1.00 [0.02 , 50.40]
✓ Romanucci 2015	1	2	1	2	1.3%	1.00 [0.02 , 50.40]
**Subtotal**		**193**		**193**	**98.2%**	**1.83 [1.24 , 2.70]**
Total events:	112		81			
Test for overall effect: Z = 3.03 (P = 0.002)						
Heterogeneity: Chi² = 21.55, df = 10 (P = 0.02); I² = 54%						
**1.6.2 Whole virus**						
✓ Basso 2019	4	5	1	5	0.5%	16.00 [0.72 , 354.80]
✓ Kim 2019	1	2	1	2	1.3%	1.00 [0.02 , 50.40]
**Subtotal**		**7**		**7**	**1.8%**	**5.29 [0.57 , 49.35]**
Total events:	5		2			
Test for overall effect: Z = 1.46 (P = 0.14)						
Heterogeneity: Chi² = 1.18, df = 1 (P = 0.28); I² = 16%						
**Total**		**200**		**200**	**100.0%**	**1.89 [1.29 , 2.78]**
Total events:	117		83			
Test for overall effect: Z = 3.25 (P = 0.001)						
Test for subgroup differences: Chi² = 0.84, df = 1 (P = 0.36), I² = 0%						
Heterogeneity: Chi² = 23.46, df = 12 (P = 0.02); I² = 49%						

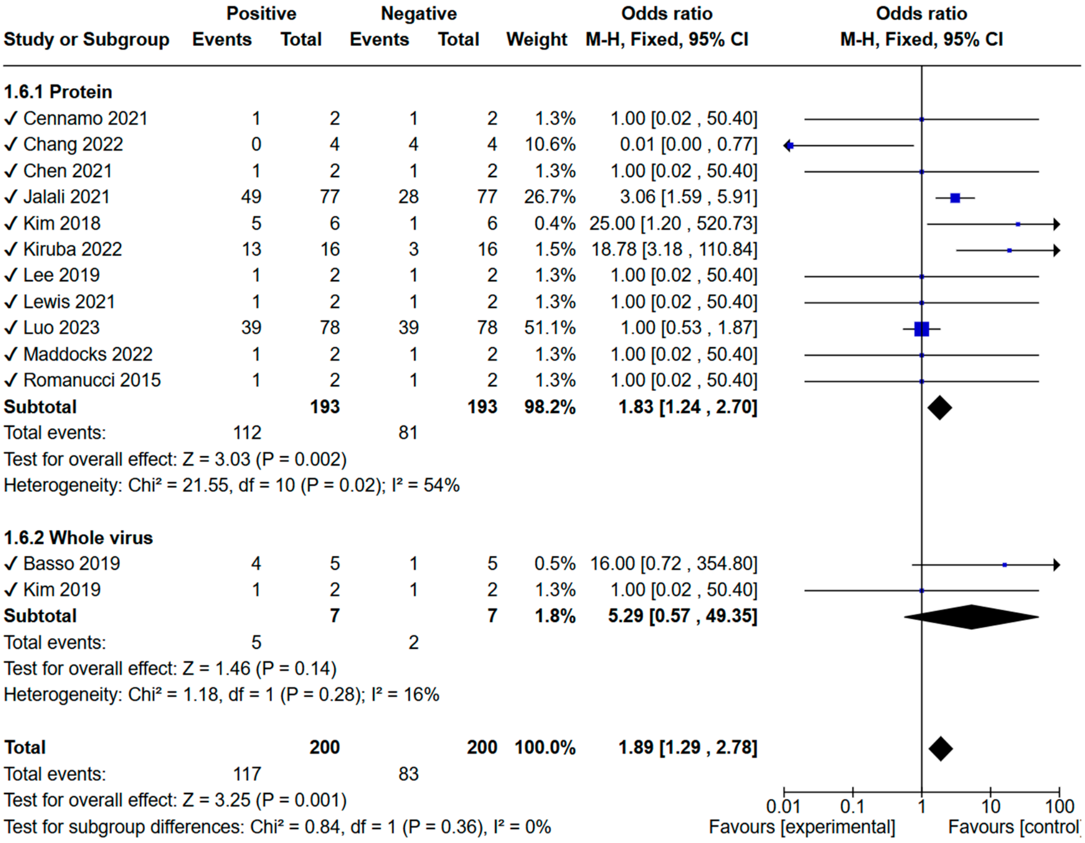

C

Study or Subgroup	Positive Events	Positive Total	Negative Events	Negative Total	Weight	Odds ratio M-H, Fixed, 95% CI
**1.3.1 SPR**						
✓ Basso 2019	4	5	1	5	0.3%	16.00 [0.72 , 354.80]
✓ Chen 2021	1	2	1	2	0.9%	1.00 [0.02 , 50.40]
✓ Jalali 2021	49	77	28	77	17.6%	3.06 [1.59 , 5.91]
✓ Kim 2018	5	6	1	6	0.3%	25.00 [1.20 , 520.73]
✓ Kim 2019	1	2	1	2	0.9%	1.00 [0.02 , 50.40]
✓ Maddocks 2022	1	2	1	2	0.9%	1.00 [0.02 , 50.40]
✓ Romanucci 2015	1	2	1	2	0.9%	1.00 [0.02 , 50.40]
**Subtotal**		**96**		**96**	**21.7%**	**3.23 [1.80 , 5.79]**
Total events:	62		34			
Test for overall effect: Z = 3.94 (P < 0.0001)						
Heterogeneity: Chi² = 4.17, df = 6 (P = 0.65); I² = 0%						
**1.3.2 LSPR**						
✓ Kim 2018	5	6	1	6	0.3%	25.00 [1.20 , 520.73]
✓ Lee 2019	1	2	1	2	0.9%	1.00 [0.02 , 50.40]
✓ Lewis 2021	1	2	1	2	0.9%	1.00 [0.02 , 50.40]
✓ Luo 2023	39	78	39	78	33.7%	1.00 [0.53 , 1.87]
**Subtotal**		**88**		**88**	**35.7%**	**1.19 [0.67 , 2.14]**
Total events:	46		42			
Test for overall effect: Z = 0.59 (P = 0.55)						
Heterogeneity: Chi² = 4.18, df = 3 (P = 0.24); I² = 28%						
**1.3.3 Fiber optic SPR**						
✓ Cennamo 2021	1	2	1	2	0.9%	1.00 [0.02 , 50.40]
✓ Chang 2022	0	4	4	4	7.0%	0.01 [0.00 , 0.77]
✓ Luo 2023	39	78	39	78	33.7%	1.00 [0.53 , 1.87]
**Subtotal**		**84**		**84**	**41.6%**	**0.83 [0.46 , 1.51]**
Total events:	40		44			
Test for overall effect: Z = 0.60 (P = 0.55)						
Heterogeneity: Chi² = 4.32, df = 2 (P = 0.12); I² = 54%						
**1.3.4 SPR colorimetric**						
✓ Kiruba 2022	13	16	3	16	1.0%	18.78 [3.18 , 110.84]
**Subtotal**		**16**		**16**	**1.0%**	**18.78 [3.18 , 110.84]**
Total events:	13		3			
Test for overall effect: Z = 3.24 (P = 0.001)						
Heterogeneity: Not applicable						
**Total**		**284**		**284**	**100.0%**	**1.66 [1.20 , 2.29]**
Total events:	161		123			
Test for overall effect: Z = 3.08 (P = 0.002)						
Test for subgroup differences: Chi² = 18.60, df = 3 (P = 0.0003), I² = 83.9%						
Heterogeneity: Chi² = 29.56, df = 14 (P = 0.009); I² = 53%						

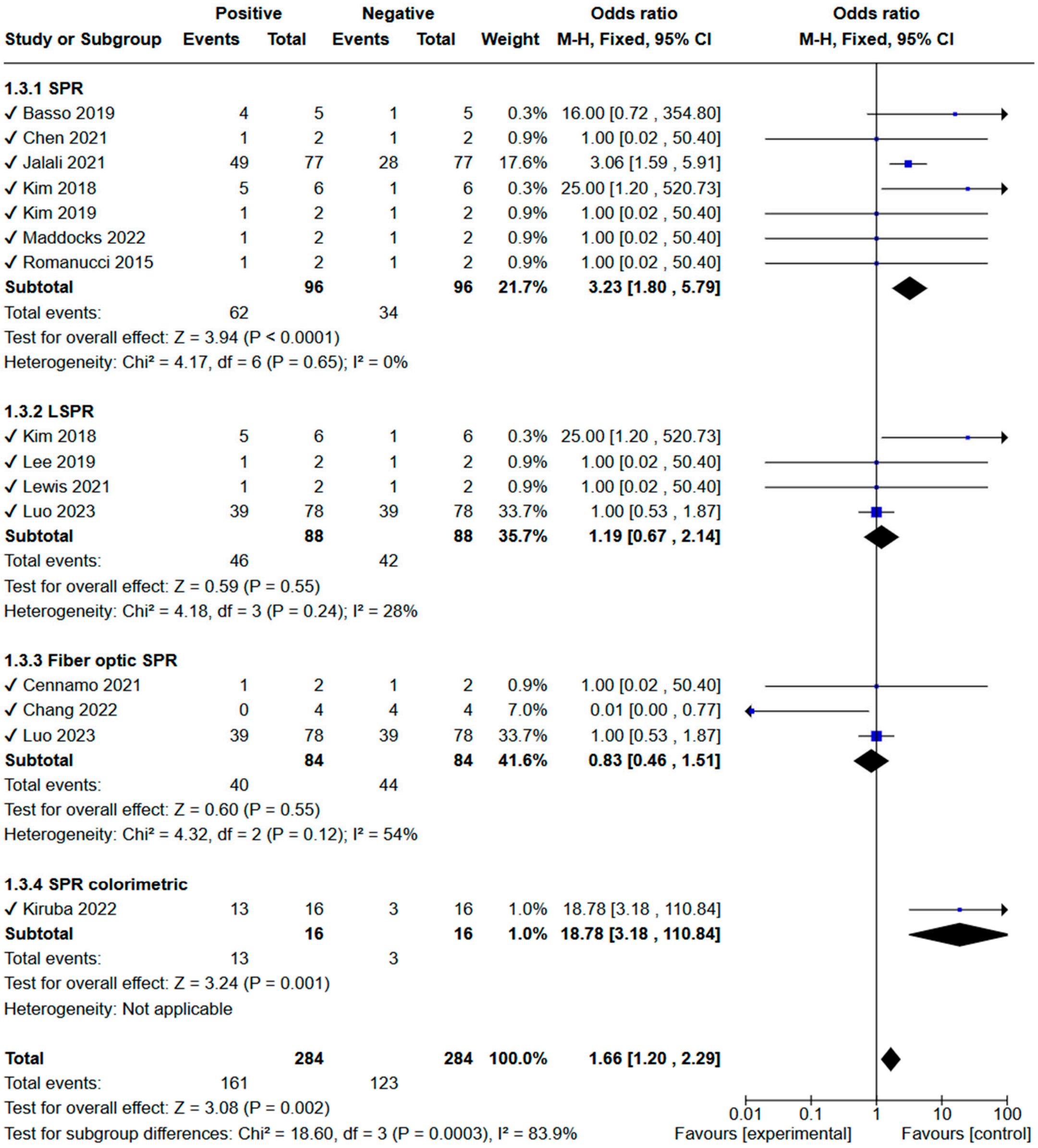

D

Study or Subgroup	Positive Events	Positive Total	Negative Events	Negative Total	Weight	Odds ratio M-H, Fixed, 95% CI
**1.5.1 Dissociation constant**						
✓ Cennamo 2021	1	2	1	2	1.3%	1.00 [0.02 , 50.40]
✓ Chang 2022	0	4	4	4	10.6%	0.01 [0.00 , 0.77]
✓ Jalali 2021	49	77	28	77	26.7%	3.06 [1.59 , 5.91]
✓ Luo 2023	39	78	39	78	51.1%	1.00 [0.53 , 1.87]
✓ Maddocks 2022	1	2	1	2	1.3%	1.00 [0.02 , 50.40]
✓ Romanucci 2015	1	2	1	2	1.3%	1.00 [0.02 , 50.40]
**Subtotal**		**165**		**165**	**92.3%**	**1.48 [0.97 , 2.27]**
Total events:	91		74			
Test for overall effect: Z = 1.82 (P = 0.07)						
Heterogeneity: Chi² = 11.48, df = 5 (P = 0.04); I² = 56%						
**1.5.2 NO dissociation contant**						
✓ Basso 2019	4	5	1	5	0.5%	16.00 [0.72 , 354.80]
✓ Chen 2021	1	2	1	2	1.3%	1.00 [0.02 , 50.40]
✓ Kim 2018	5	6	1	6	0.4%	25.00 [1.20 , 520.73]
✓ Kim 2019	1	2	1	2	1.3%	1.00 [0.02 , 50.40]
✓ Kiruba 2022	13	16	3	16	1.5%	18.78 [3.18 , 110.84]
✓ Lee 2019	1	2	1	2	1.3%	1.00 [0.02 , 50.40]
✓ Lewis 2021	1	2	1	2	1.3%	1.00 [0.02 , 50.40]
**Subtotal**		**35**		**35**	**7.7%**	**6.80 [2.41 , 19.20]**
Total events:	26		9			
Test for overall effect: Z = 3.62 (P = 0.0003)						
Heterogeneity: Chi² = 5.93, df = 6 (P = 0.43); I² = 0%						
**Total**		**200**		**200**	**100.0%**	**1.89 [1.29 , 2.78]**
Total events:	117		83			
Test for overall effect: Z = 3.25 (P = 0.001)						
Test for subgroup differences: Chi² = 7.10, df = 1 (P = 0.008), I² = 85.9%						
Heterogeneity: Chi² = 23.46, df = 12 (P = 0.02); I² = 49%						

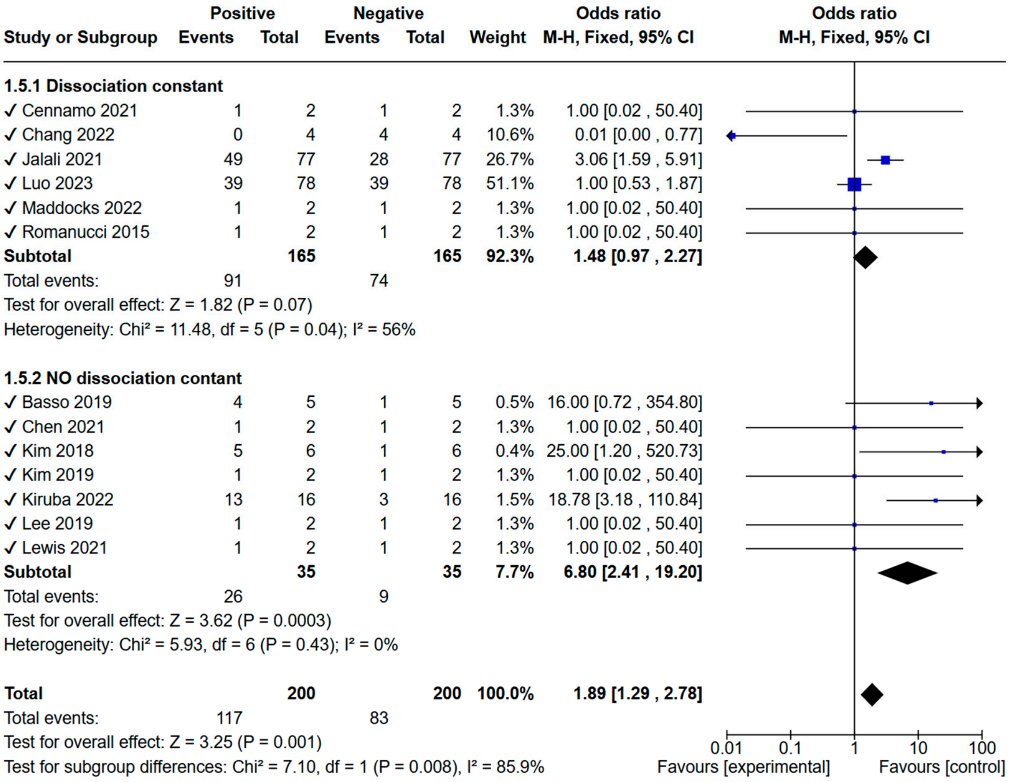

E

Study or Subgroup	Positive Events	Positive Total	Negative Events	Negative Total	Weight	Odds ratio M-H, Fixed, 95% CI
**1.4.1 Reference**						
✓ Basso 2019	4	5	1	5	0.5%	16.00 [0.72 , 354.80]
✓ Cennamo 2021	1	2	1	2	1.3%	1.00 [0.02 , 50.40]
✓ Chang 2022	0	4	4	4	10.6%	0.01 [0.00 , 0.77]
✓ Kim 2018	5	6	1	6	0.4%	25.00 [1.20 , 520.73]
✓ Kim 2019	1	2	1	2	1.3%	1.00 [0.02 , 50.40]
✓ Lee 2019	1	2	1	2	1.3%	1.00 [0.02 , 50.40]
✓ Maddocks 2022	1	2	1	2	1.3%	1.00 [0.02 , 50.40]
**Subtotal**		**23**		**23**	**16.8%**	**1.47 [0.54 , 4.00]**
Total events:	13		10			
Test for overall effect: Z = 0.75 (P = 0.45)						
Heterogeneity: Chi² = 10.92, df = 6 (P = 0.09); I² = 45%						
**1.4.2 NO Reference**						
✓ Chen 2021	1	2	1	2	1.3%	1.00 [0.02 , 50.40]
✓ Jalali 2021	49	77	28	77	26.7%	3.06 [1.59 , 5.91]
✓ Kiruba 2022	13	16	3	16	1.5%	18.78 [3.18 , 110.84]
✓ Lewis 2021	1	2	1	2	1.3%	1.00 [0.02 , 50.40]
✓ Luo 2023	39	78	39	78	51.1%	1.00 [0.53 , 1.87]
✓ Romanucci 2015	1	2	1	2	1.3%	1.00 [0.02 , 50.40]
**Subtotal**		**177**		**177**	**83.2%**	**1.98 [1.31 , 2.99]**
Total events:	104		73			
Test for overall effect: Z = 3.22 (P = 0.001)						
Heterogeneity: Chi² = 12.76, df = 5 (P = 0.03); I² = 61%						
**Total**		**200**		**200**	**100.0%**	**1.89 [1.29 , 2.78]**
Total events:	117		83			
Test for overall effect: Z = 3.25 (P = 0.001)						
Test for subgroup differences: Chi² = 0.29, df = 1 (P = 0.59), I² = 0%						
Heterogeneity: Chi² = 23.46, df = 12 (P = 0.02); I² = 49%						

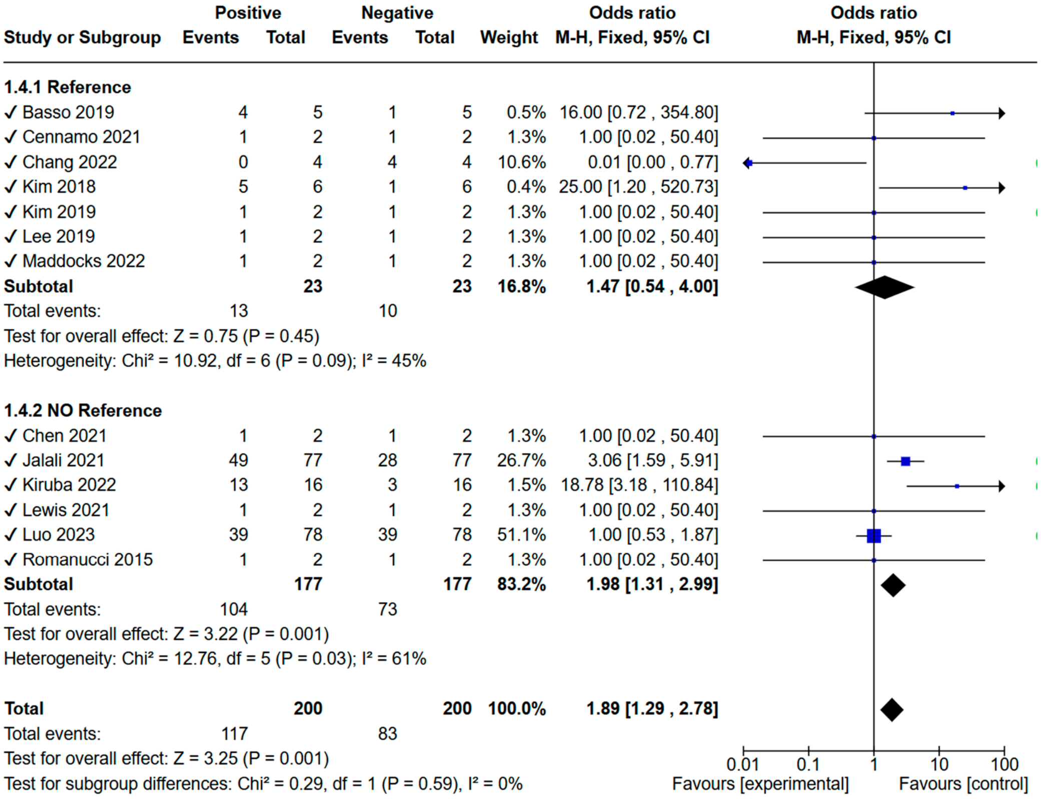

Figure S1. Subgroup analysis for pooled diagnostic odds ratio based on the type of virus (A), virus target (B), instrument configuration (C), kinetics (D) reference method (E).
